# Comparison of Common Algorithms for Single-Pixel Imaging via Compressed Sensing

**DOI:** 10.3390/s23104678

**Published:** 2023-05-11

**Authors:** Wenjing Zhao, Lei Gao, Aiping Zhai, Dong Wang

**Affiliations:** 1 College of Physics and Optoelectronics, Taiyuan University of Technology, No. 79 West Main Street, Taiyuan 030024, China; zhaowenjing01@tyut.edu.cn (W.Z.); zggaoleigg@gmail.com (L.G.); zhaiaiping@tyut.edu.cn (A.Z.); 2 Key Laboratory of Advanced Transducers and Intelligent Control System, Ministry of Education, and Shanxi Province, Taiyuan University of Technology, No. 79 West Main Street, Taiyuan 030024, China

**Keywords:** compressed sensing, optical signal processing, single-pixel imaging

## Abstract

Single-pixel imaging (SPI) uses a single-pixel detector instead of a detector array with a lot of pixels in traditional imaging techniques to realize two-dimensional or even multi-dimensional imaging. For SPI using compressed sensing, the target to be imaged is illuminated by a series of patterns with spatial resolution, and then the reflected or transmitted intensity is compressively sampled by the single-pixel detector to reconstruct the target image while breaking the limitation of the Nyquist sampling theorem. Recently, in the area of signal processing using compressed sensing, many measurement matrices as well as reconstruction algorithms have been proposed. It is necessary to explore the application of these methods in SPI. Therefore, this paper reviews the concept of compressive sensing SPI and summarizes the main measurement matrices and reconstruction algorithms in compressive sensing. Further, the performance of their applications in SPI through simulations and experiments is explored in detail, and then their advantages and disadvantages are summarized. Finally, the prospect of compressive sensing with SPI is discussed.

## 1. Introduction

In traditional imaging using silicon-based charge-coupled devices (CCD) or complementary metal-oxide-semiconductor (CMOS) array detectors, the imaging resolution is proportional to the number of pixels on the detector. Thus, to improve the imaging resolution, the integration of the detector needs to be increased, which leads to higher requirements for manufacturing. Single-pixel imaging (SPI) [1,2] uses a single-pixel detector, instead of a detector array with a lot of pixels in traditional imaging, to realize two-dimensional or even multi-dimensional imaging, which gives cost-efficiency to broadband imaging and resolution enhancement, and recently has a lot of attention with the potential of applications in various imaging areas such as visible range imaging [3], multispectral imaging [4,5,6,7], hyperspectral imaging [8], ultrafast imaging [9], infrared imaging [10], terahertz imaging [11,12,13], gas imaging [14], microscopic imaging [15,16,17], imaging through scattering media [18,19], photoacoustic imaging [20], long-range imaging [21,22], X-ray imaging [23,24], 3D imaging [25] and holography [26], etc.

For SPI, the target to be projected is modulated by a series of patterns with spatial resolution, and then the reflected or transmitted intensity is collected by the single-pixel detector to reconstruct the target image. The modulation patterns can be realized by a diffuser, a spinning mask, or a spatial light modulator. The single-pixel detector can be a photodiode, a photon multiplier, or a conventional image detector used as a bucket detector. SPI can be traced back to quantum ghost imaging and thermal ghost imaging. According to the position of the modulation device, SPI can be divided into computational ghost imaging (CGI, which uses active illumination) and single-pixel cameras (SPC, which uses passive detection) [27,28]. Despite being commonly treated as separate research fields, it has become obvious that, from an optical perspective, CGI and SPC are the same. Therefore, in this paper, we uniformly call it SPI.

SPI can be divided into orthogonal SPI [29,30,31,32,33] and compressed sensing SPI (CSSPI) [1,34,35,36,37]. Firstly, orthogonal SPI often seeks to solve an inverse problem or perform a reconstruction from an ensemble average. Secondly, CSSPI often seeks the sparse estimation of an optimization problem. CSSPI was first realized by Duarte et al. [1]. The target to be imaged is modulated by a series of random patterns. Then, the reflected or transmitted intensity is compressively sampled by the single-pixel detector, which reduces the measurement number. Finally, the target image is reconstructed using compressed sensing (CS) theory.

The CS theory was proposed in 2006 [38,39], which holds that it is possible to recover the original signal from under-sampled data when the original target signal is sparse or is sparse in the transform domain. This breaks the limitation of the Nyquist sampling theorem in data acquisition and thus reduces the sampling rate.

CS mainly focuses on how to obtain the whole information of the target signal by sampling the data much less than the Nyquist sampling method does, as well as how to recover the target signal from the down-sampled data. Recently, on these two key issues, researchers have developed a variety of sampling frameworks, i.e., different measurement matrices, and a variety of signal reconstruction algorithms. It is necessary to explore the application of these methods in SPI.

Additionally, refs. [40,41] review the development of CS; refs. [42,43] review the main measurement matrices in CS; and refs. [44,45,46] review the reconstruction algorithms in CS. In addition, refs. [2,47] review the development of SPI. Refs. [48,49] review the algorithms of SPI; however, for CS, only the TVAL3 algorithm is involved. There are no reviews on compressive sensing SPI and, especially, no detailed investigations on the performance of SPI using these different CS methods. As a result, this paper reviews the concept of CSSPI and summarizes the main measurement matrices and reconstruction algorithms. Further, their performance is explored in detail through simulations and experiments, and then their advantages and disadvantages are summarized. Finally, the prospect of CSSPI is discussed. Table 1 shows the comparison between the works of this paper and the existing works.

The paper is structured as follows: In Section 2, we review the principles of CSSPI and point out that the measurement matrix and reconstruction algorithm are the two important factors affecting its performance. In Section 3, we classify some main measurement matrices and briefly introduce how to generate them. In Section 4, we classify the existing CS reconstruction algorithms based on sparse and image gradient sparse and review their principles of reconstruction. Section 5 and Section 6 compare the performance of these measurement matrices and reconstruction algorithms through simulations and experiments, respectively. Finally, Section 7 summarizes the work of this paper, and discussions on the prospect of CSSPI are given.

## 2. Compressed Sensing SPI

**Figure 1:**
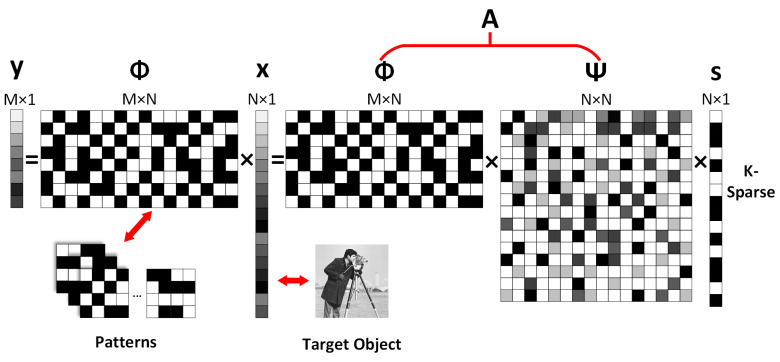
Principle of CSSPI.

As shown in Figure 1, assuming that a target object with a spatial resolution of u×v pixels is to be captured by the CSSPI, the imaging process is comprised of two procedures. The first procedure is to modulate the object with a set of patterns with a spatial resolution of u×v pixels, and measure the corresponding reflected light $y\in {\mathbb{R}}^{M\times 1}$, which can be mathematically described as,

(1) y=Φx,

where each row of the measurement matrix $\Phi \in {\mathbb{R}}^{M\times N}$ represents a 2-D modulation pattern in its 1-D representation, $x\in {\mathbb{R}}^{N\times 1}$ is the signal of the 2-D target object in its 1-D representation, N=u×v is the length of the 1-D representations, and *M* is the number of measurements and also equals the number of the modulation patterns.

The second procedure is to reconstruct the image of the target object using CS [50,51]. Normally, the number of measurements is less than the length of the signal, i.e., M<N, which is demanded. This makes solving the signal *x* from Equation (1) an undetermined question. Fortunately, CS suggests that when the signal *x* is sparse or has some sparse representation in some sparse dictionaries, e.g., discrete cosine transform (DCT) and wavelet transform (WT) [52,53,54,55,56], the joint matrix is composed of the measurement matrix, and the sparse dictionary satisfies the restricted isometry property (RIP) [38,57], it is possible to reconstruct the original signal x [58]. As shown in Figure 1, using Ψs to replace x in Equation (1) as the object vector, the equivalent form of Equation (1) can be written:

(2) y=Φx=ΦΨs=As,

where $\Psi \in {\mathbb{R}}^{N\times N}$ is the sparse dictionary, $A\in {\mathbb{R}}^{M\times N}$ is the joint matrix, and $s\in {\mathbb{R}}^{N\times 1}$ is the sparse vector. In this case, solving the signal x from Equations (1) and (2) can be transformed into an optimization problem for solving *l_0_*-norm minimization [39,59]:

(3) $$\underset{\hat{s}\in {\mathbb{R}}^{n}}{\min}{\left\|\hat{s}\right\|}_{l_{0}}\;\mathrm{subject}\;\mathrm{to}\;A\hat{s}=y.$$

In order to solve the optimization problem of Equation (3), there are two requirements. First, the matrix A that satisfies the RIP of the following equation is demanded.

(4) $$(1-\delta ){\left\|s\right\|}_{2}^{2}\leq {\left\|As\right\|}_{2}^{2}\leq (1+\delta ){\left\|s\right\|}_{2}^{2},$$

where δ∈(0,1) is the restricted isometry constant (RIC) value of the matrix A. In practical use, the random matrix is a method to obtain joint matrices following RIP conditions, however, it is difficult to verify whether these matrices satisfy the RIP property with a low RIC value. Most of the time, it only needs to ensure that the coherence between the measurement matrix and the sparse dictionary [60] is minimized. The correlation between measurement matrix Φ and sparse dictionary Ψ is defined as: $\mu (\Phi ,\Psi )=\sqrt{N}\underset{1\leq k,j\leq N}{\max}\left|\langle \varphi_{{}_{k}},\psi_{j}\rangle \right|$. To put it simply, it is to find the maximum correlation between the two matrix elements coherently. If there are correlation elements between Φ and Ψ, the correlation is very large, otherwise, the correlation is very small. There are three types of measurement matrices for CSSPI [46]: random measurement matrices, partial orthogonal measurement matrices, and semi-deterministic random measurement matrices. Second, finding a suitable reconstruction algorithm to solve the optimization problem of Equation (3) is necessary. Over the past years, several typical sparse recovery algorithms have been proposed [50], which can be classified into five main categories: convex optimization algorithms, greedy algorithms, non-convex optimization algorithms, Bregman distance minimization algorithms, and total variation minimization algorithms. Comments on the measurement matrices and the reconstruction algorithms will be made in Section 3 and Section 4, respectively.

## 3. Selection of Measurement Matrix

As mentioned in Section 2, to achieve CSSPI, it is necessary to select an appropriate measurement matrix, i.e., the matrix that satisfied the RIP or the coherence between and is small, to encode the object. There are three types of measurement matrices for compressive sensing SPI [46]: random measurement matrices [47,61,62,63], partial orthogonal measurement matrices [47,64,65,66], and semi-deterministic random measurement matrices [64,67,68,69,70,71,72,73,74,75,76]. In addition, there are some optimized measurement matrices proposed for improving the RIP characteristics of the measurement matrix [77,78,79,80,81,82,83,84,85] or machine learning [86,87,88,89].

### 3.1. Random Measurement Matrix

Each element of the random measurement matrix is independent and obeys the same distribution, e.g., Gaussian distribution, Bernoulli distribution, etc. It has been proven in [61] that such matrices have no coherence with most sparse signals and sparse dictionaries; that is, a small number of measurements are needed to accurately reconstruct the target object. However, such matrices almost do not exist in reality and can only be generated in the laboratory. At the same time, the drawbacks of the high computational complexity and large storage space of the reconstructed object limit its use in practice. Common random measurement matrices include the Gaussian random measurement matrix [62], the Bernoulli random measurement matrix [63], etc.

#### 3.1.1. Gaussian Random Measurement Matrix

A Gaussian random measurement matrix is the most widely used measurement matrix in compressed sensing, which constructs a Gaussian distribution matrix in which each element independently obeys the mean of 0 and the variance of *M*. That is:

(5) $$\phi_{i,j}\sim N\left(0,\frac{1}{M}\right).$$

Each element of the matrix is distributed independently and has strong randomness, which is not related to most sparse signals and sparse bases. When the Gaussian random matrix is used to measure the signal, if the number of measured values satisfies $M\geq ck\log\frac{N}{k}$, the RIP condition (c is a constant) is greatly satisfied, and the signal is reconstructed accurately.

#### 3.1.2. Bernoulli Random Measurement Matrix

The difference between the Bernoulli random measurement matrix and the Gaussian random measurement matrix is that each element in the matrix is independent and uniformly distributed and obeys the binomial Bernoulli distribution with a probability of 1/2, that is:

(6) $$\phi_{i,j}=\{\begin{array}{c} 1P=\frac{1}{2}\\ -1P=\frac{1}{2} \end{array}.$$

Similar to the Gaussian random matrix, each element of the matrix is independently distributed, which also has strong randomness and is not related to most sparse signals and sparse bases. Additionally, since the matrix elements are only 1 and −1, it is easy to implement on hardware devices; hence, it is more widely used than a Gaussian random matrix in practical applications.

### 3.2. Partial Orthogonal Measurement Matrix

Given the drawbacks of high computational complexity, large storage space, and high uncertainty of the random measurement matrices, it is particularly important to find or design a deterministic measurement matrix to reduce the computational complexity and storage space. The partial orthogonal matrix is derived from the existing orthogonal matrix with some special properties in the field of signal processing.

#### 3.2.1. Partial Hadamard Matrix

In refs. [65,66], the partial Hadamard matrix is proposed as the measurement matrix of CS. It is mainly composed of M-row vectors in the N×N Hadamard matrix. The entries of the Hadamard matrix are 1 and −1, and its columns are orthogonal. The matrix satisfies the following properties:

(7) $$HH^{T}=NI_{N},$$

where *I_N_* is the N×N identity matrix and *H^T^* is the transpose of the matrix *H*. This measurement matrix is incoherent for most sparse signals and sparse dictionaries. However, since the order of the Hadamard matrix must satisfy 2*n*, n=1,2,3,⋯, there are strict requirements for the dimension of the target signal, which limits its use in practice.

#### 3.2.2. Partial Fourier Matrix

In addition, the partial Fourier matrix is proposed as the measurement matrix of CS in Refs [47]. It can reduce the complexity of the algorithm by using a fast Fourier transform. However, it is usually only incoherent to time-domain sparse signals, and most natural images do not meet this condition, so this kind of matrix is rarely used in CSSPI.

### 3.3. Semi-Deterministic Random Measurement Matrix

The semi-deterministic random measurement matrices are designed to follow a deterministic construction to satisfy the RIP or to have low mutual coherence, which can be regarded as a combination of random measurement matrices and deterministic orthogonal measurement matrices. At present, this kind of matrix mainly includes Toeplitz and circulant matrices [67,68], structured random matrices [68,69,72], sparse random matrices [70,71,72,73], binary random matrices [74], block-diagonal matrices [75,76], etc.

#### 3.3.1. Toeplitz and Circulant Matrix

The Toeplitz and circulant measurement matrices are generated based on random measurement matrices and have the following forms:

(8) $$T=\left(\begin{array}{cccc} t_{n} & t_{n-1} & \cdots & t_{1}\\ t_{n+1} & t_{n} & \cdots & t_{2}\\ \vdots & \vdots & \ddots & \vdots \\ t_{2n-1} & t_{2n-2} & \cdots & t_{n} \end{array}\right)\;\mathrm{and}\;C=\left(\begin{array}{cccc} t_{n} & t_{n-1} & \cdots & t_{1}\\ t_{1} & t_{n} & \cdots & t_{2}\\ \vdots & \vdots & \ddots & \vdots \\ t_{n-1} & t_{n-2} & \cdots & t_{n} \end{array}\right),$$

where the diagonal elements of each matrix are the same (*T_i,j_ = T_i+1,j+1_*). A circulant matrix is a special form of the Toeplitz matrix. The elements in the first row of the matrix obey the same random distribution as the random measurement matrix.

#### 3.3.2. Sparse Random Matrix

The structure of the sparse random matrix is simple, and it is easy to generate and save in the experiment. The elements of d random positions in each column are 1, and the rest are all 0, and d∈{4,8,10,16} [72].

In summary, the three types of measurement matrices have their advantages and disadvantages. The random measurement matrices are close to the characteristics of RIP to a great extent; however, they are not easy to generate in reality and require high computational complexity and storage capacity. Part of the orthogonal measurement matrices has a very low computational complexity according to its orthogonal transformation, but it also has some limitations in its transformation. The semi-deterministic random measurement matrices combine the properties of random measurement matrices and partial orthogonal measurement matrices to some extent. Table 2 shows the advantages and disadvantages of each measurement matrix in detail.

The measurement matrices constructed by different construction methods are proposed [77,78,79,80,81,82,83]. Researchers try to improve the RIP property of the existing measurement matrix. David L. Donoho proposed in Ref. [61] that the minimum singular value of the submatrix composed of the column vectors of the measurement matrix must be greater than a positive constant, i.e., the column vectors of the matrix satisfy certain independence. The QR decomposition of a matrix can increase the singular value of the matrix without changing its properties. In addition, methods for matrix optimization are also proposed, such as an optimal projection matrix that optimizes the measurement matrix by using the sparse dictionary of signals [84] and an optimal measurement matrix based on effective projection [85]. Researchers are also trying to use machine learning to train and optimize the sampling framework of CS [86,87,88,89].

## 4. Selection of the Reconstruction Algorithm

The previous section gives requirements for the measurement matrices Φ, so that x can be recovered from the given measurements y=Φx=ΦΨs=As. Since knowledge of s is equivalent to knowledge of x. Here, we only need to discuss the reconstruction algorithms for solving the equation y=As.

As mentioned in Section 2, the unique solution of Equation (2) can be obtained by posing the reconstruction problem as an $l_{0}$-minimization problem given by Equation (3). However, solving the $l_{0}$-minimization program is an NP-complete problem. Therefore, in practice, it is unable to solve it. The crux of CS is to propose faster algorithms that can solve Equation (3) with high probability. Over the past years, several typical sparse recovery algorithms have been proposed [50,90,91,92,93], which can be classified into five main categories: convex optimization algorithms, greedy algorithms, non-convex optimization algorithms, Bregman distance minimization algorithms, and total variation minimization algorithms.

### 4.1. Convex Optimization Algorithms

The algorithms reduce the CS reconstruction problem to a convex optimization problem of Equation (9) [94,95,96,97,98,99,100], which can be solved using methods of linear programming.

(9) $$\underset{\hat{s}\in {\mathbb{R}}^{n}}{\min}{\left\|\hat{s}\right\|}_{l_{1}}\;\mathrm{subject}\;\mathrm{to}\;As=y.$$

#### 4.1.1. Basis Pursuit

Basis pursuit (BP) is a signal processing technique [101,102], which aims to decompose a signal into a superposition of dictionary elements that have the smallest $l_{0}$-norm of the coefficients, subject to the equality constraint given in Equation (9). Since BP is based on global optimization, it can be solved stably in many ways. Especially, it is a principle of global optimization without any specified algorithm, which is closely connected with linear programming. Therefore, Equation (9) can be expressed as:

(10) $$\min\;c^{T}\hat{s}\;\mathrm{subject}\;\mathrm{to}\;O\;\hat{s}=y;\;\hat{s}\geq 0,$$

where $c^{T}\;\hat{s}$ is the objective function, $\;\hat{s}\geq 0$ is a set of bounds, *O* = (*A, -A*), and *c =* (1,1). Over the past few decades, many algorithms have been proposed to solve linear programming problems, such as the simplex method and interior point method [103].

#### 4.1.2. Basis Pursuit Denoising/Least Absolute Shrinkage and Selection Operator

If the measurements are corrupted by noise in CSSPI, strategies for noise suppression must be sought. Based on the principle of global optimization of BP, the widely used algorithms for robust data recovery from noisy measurements are basis pursuit denoising (BPDN) [101] and least absolute shrinkage and selection operator (LASSO) [104,105,106], etc. The BPDN and LASSO algorithms consider the sparse estimation problem as shown in Equation (11):

(11) $$\min{\left\|\hat{s}\right\|}_{1}\;s.t.\;y=As+e,$$

where e denotes noise and s is a pure signal without noise contamination. The system can be translated into the following optimization problem:

(12) $$\underset{s}{\min}\frac{1}{2}{\left\|y-A\hat{s}\right\|}_{2}^{2}+\lambda {\left\|\hat{s}\right\|}_{1},$$

where λ is a scalar parameter, which determines the magnitude of the signal estimation residual and has a great impact on the performance of BPDN and LASSO algorithms. As λ→0 the residual goes to zero, this problem translates into the same problem as BP. As λ→∞ the residual gets larger, the measurements are completely drowned out by noise. In ref. [101], the author suggests that λ can be set as $\lambda_{p}=\sigma \sqrt{2\log(p)}$, where σ is the level of noise and p is the number of dictionary bases.

#### 4.1.3. Decoding by Linear Programming

Ref. [107] proposed a faster method to solve sparse solutions of underdetermined equations: decoding by linear programming (DLP). It is similar to BPDN and LASSO, which consider the existence of noise in CSSPI. To recover s from corrupted data y=As+e, DLP considers solving the following *l_1_*-minimization problem:

(13) $$\underset{g}{\min}{\left\|y-Ag\right\|}_{l_{1}},$$

where g is the estimate and s is the unique solution of Equation (13). Equation (13) is a linear programming with inequality constraints and can be solved efficiently using standard optimization algorithms, see ref. [108].

#### 4.1.4. Dantzig Selector

Dantzig selector (DS) is another solver for CS [109], which estimates the target signal s from measurements contaminated by noise. DS considers solving the convex optimization problem:

(14) $$\min{\left\|\hat{s}\right\|}_{1}\;s.t.\;{\left\|A^{T}r\right\|}_{\infty }\leq \sqrt{1+\delta_{1}}\lambda_{N}\cdot \sigma ,$$

where $r=y-A\hat{s}$ is the residual vector, σ is the standard deviation of the additive white Gaussian noise, $\lambda_{N}>0$ and $\sqrt{1+\delta_{1}}$ is the maximum Euclidean norm of A. In addition, the program DS can easily be recast as linear programming.

The toolbox proposed in ref. [108] uses a primal-dual algorithm to solve all kinds of linear programming transformed from convex optimization problems.

### 4.2. Greedy Algorithms

Greedy algorithms [110,111,112] are the second class of CS reconstruction algorithms. These algorithms are different from the convex optimization algorithms, which try to find the global optima; they try to find the best local optima in the immediate neighborhood for each iteration of the optimization.

#### 4.2.1. Orthogonal Matching Pursuit

Orthogonal matching pursuit (OMP) computes the best nonlinear approximation for the sparse solution of Equation (3), which is proposed by Y. C. Pati et al. [113,114,115,116,117]. By taking the higher absolute value of the inner product calculated between each column and the residue, it locates the column in the matrix A with the largest correlation to the residue $r=y-A\hat{s}$. In addition, OMP fits the original function to all the already selected dictionary elements via least squares or projects the function orthogonally onto all selected dictionary atoms, so it does not repeatedly select the same atom.

OMP has become one of the most widely used CS algorithms in recent years. A couple of improved OMPs have been proposed, such as stagewise orthogonal matching pursuit (StOMP) and regularized orthogonal matching pursuit (ROMP) [118,119].

#### 4.2.2. Compressive Sampling Matching Pursuit/Subspace Pursuit

Each iteration of OMP selects only one atom, and is also called the serial greedy algorithm. To overcome the instability of serial greedy algorithms, researchers propose parallel greedy algorithms, i.e., compressive sampling matching pursuit (CoSaMP) [120] and subspace pursuit (SP) [121]. This kind of algorithm has stricter limits on convergence and performance, selecting multiple atoms once in each iteration and allowing the previously selected wrong atoms to be discarded. CoSaMP selects 2k atoms, and SP selects k atoms in each iteration.

#### 4.2.3. Iterative Hard Thresholding

Iterative hard thresholding (IHT) is yet another greedy algorithm that was proposed by Blumensath and Davies [122,123,124]. It introduces the thresholding function in each iteration to maintain the k maximum non-zero entries in the estimated signal, and the remaining entries are set to zero.

The critical update step of IHT is,

(15) $$s^{\left(n+1\right)}=H_{k}(s^{\left(n\right)}+\lambda A^{T}(y-As^{\left(n\right)})),$$

where *H_k_* is the hard thresholding function and λ denotes the step size.

### 4.3. Non-Convex Optimization Algorithms

All CS reconstruction algorithms based on signal sparsity try to find the approximate solution that satisfies the minimum norm. Non-convex optimization algorithms recover signals from fewer measurements by replacing *l_1_*-norm with *l_p_*-norm where p≤1 [125,126,127].

#### 4.3.1. Iterative Reweighted Least Square Algorithm

The equivalent variant form of Equation (9) is considered in the non-convex optimization algorithm:

(16) $$\underset{\hat{s}\in {\mathbb{R}}^{n}}{\min}{\left\|\hat{s}\right\|}_{l_{p}}\;\mathrm{subject}\;\mathrm{to}\;As=y,$$

where p<1. The iterative reweighted least squares algorithm (IRLS) [128,129] is to replace the *l_p_* objective function in (16) with a weighted *l_2_*-norm:

(17) $$\underset{s}{\min}\sum_{i=1}^{N}\omega_{i}s_{i}^{2}\;\mathrm{subject}\;\mathrm{to}\;As=y,$$

where the weights are computed from the previous iterate *s*^(*n*−1)^ so that the objective in (17) is a first-order approximation $\omega_{i}={\left|s_{i}^{\left(n-1\right)}\right|}^{p-2}$. The solution of (17) can be given explicitly, giving the next iteration *s*^(*n*)^:

(18) $$s^{\left(n\right)}=Q_{n}A^{T}{\left(AQ_{n}A^{T}\right)}^{-1}y,$$

where *Q_n_* is the diagonal matrix with entries $1/\omega_{i}={\left|s_{i}^{\left(n-1\right)}\right|}^{2-p}$.

#### 4.3.2. Bayesian Compressed Sensing Algorithm

The Bayesian approach applies to the input signals, which belong to some known probability distributions. The Bayesian compressed sensing algorithm (BCS) [130,131,132,133] provides an estimate of the posterior density function of the additive noise encountered when performing compressive measurements, e.g., y=As+n. Let $\sigma^{2}$ be the noise variance; there is a Gaussian likelihood model:

(19) $$p(y|s,\sigma^{2})={\left(2\pi \sigma^{2}\right)}^{-\frac{k}{2}}\exp(-\frac{1}{2\sigma^{2}}{\left\|y-As\right\|}^{2}).$$

In this way, the traditional sparse weight inversion problem of CS is transformed into a linear regression problem with sparse constraints, and the BCS seeks a complete posterior density function.

### 4.4. Bregman Distance Minimization Algorithms

For the large-scale and completely dense matrix *A*, the *As* and *A^T^s* can be calculated by fast transformation, which makes it possible to solve the unconstrained problem:

(20) $$\underset{s}{\min}\mu {\left\|s\right\|}_{1}+\frac{1}{2}{\left\|y-As\right\|}_{2}^{2}.$$

By iteratively solving a series of unconstrained subproblems shown in Equation (20) generated by the Bregman iterative regularization scheme, the exact solution of the constrained problem is given [134,135,136].

In [137], the split Bregman (SB) algorithm has been proposed, which can be used in CS. Due to its parallelizing nature, it can be efficiently implemented to have faster computation.

### 4.5. Total Variation Minimization Algorithms

For two-dimensional image signals, Rudin, Osher, and Fatemi [138] first introduced the concept of total variation (TV) for image denoising in 1992. As a result, a new restoration model is proposed that is based on the sparse image gradient. Research has confirmed that the use of TV minimization in CS makes the recovered image quality sharper by preserving the edges or boundaries more accurately, which is essential to characterizing images. Different from other algorithms, the TV minimization algorithms do not need a specific sparse dictionary to represent image signals. A detailed discussion of TV minimization algorithms can be found in [139], and different versions of the TV minimization algorithm for image restoration are proposed in [140,141,142].

Definition: Let *x_ij_* denote the pixel in the *i*-th row and *j* column of an n×n image, and define the operators

(21) $$D_{h;ij}x=\{\begin{array}{cc} x_{i+1,j}-x_{ij}\; & i<n\\ 0 & i=n \end{array}\;,\;D_{v;ij}x=\{\begin{array}{cc} x_{i,j+1}-x_{ij}\; & j<n\\ 0\; & j=n \end{array},$$

and

(22) $$D_{ij}x=\left(\begin{array}{c} D_{h;ij}x\\ D_{v;ij}x \end{array}\right),$$

where the 2-vector *D_ij_x* can be interpreted as a kind of discrete gradient of the digital image x. The total variation of x is simply the sum of the magnitudes of this discrete gradient at every point:

(23) $$TV(x)=\sum_{ij}\sqrt{{\left(D_{h;ij}x\right)}^{2}+{\left(D_{v;ij}x\right)}^{2}}=\sum_{ij}{\left\|D_{ij}x\right\|}_{2}.$$

#### 4.5.1. Min-TV with Equality Constraints

If *D_ij_x* is nonzero for only a small number of indices ij for the image x, the image signal can be restored by solving the following equality constraint problems, which are called min-TV with equality constraints (TV-EQ):

(24) min TV(x) subject to Φx=y.

#### 4.5.2. Min-TV with Quadratic Constraints

Additionally, for the image signal with a gradient sparse property (i.e., only a small amount of *D_ij_x* is non-zero) and the measured value of the single pixel is polluted by noise, the equality constraint problem of Equation (24) can be transformed into the following min-TV with quadratic constraints (TV-QC) problem:

(25) $$\min\;TV(x)\;\mathrm{subject}\;\mathrm{to}\;{\left\|\Phi x-b\right\|}_{2}\leq \varepsilon .$$

#### 4.5.3. TV Dantzig Selector

The solver of the DS proposed in [109] can also solve the TV minimization of the CSSPI problem when considering noise pollution. The TV Dantzig selector (TV-DS) considers the following TV minimization issues:

(26) $$\min\;TV(x)\;\mathrm{subject}\;\mathrm{to}\;{\left\|\Phi^{*}(\Phi x-b)\right\|}_{\infty }\leq \gamma .$$

#### 4.5.4. Total Variation Augmented Lagrangian Alternating Direction Algorithm

Compared with the traditional convex optimization algorithms based on signal sparsity, the above three kinds of TV minimization algorithms are still much slower (e.g., TV-DS) or have poor image reconstruction quality (e.g., TV-QC). The total variation augmented Lagrangian alternating direction algorithm (TVAL3) has successfully overcome this difficulty and accepts a vast range of measurement matrices [142].

The TV minimization model is very difficult to solve directly due to the non-differentiability and non-linearity of the TV term. TVAL3 minimizes augmented Lagrangian functions through an alternating minimization scheme and updates multipliers after each sweep. Instead of employing the augmented Lagrangian method to minimize Equation (23) directly, consider an equivalent variant of Equation (23):

(27) $$\underset{\omega_{i},x}{\min}\sum_{i}\left\|\omega_{i}\right\|,\;\mathrm{subject}\;\mathrm{to}\;\Phi x=y\;\mathrm{and}\;D_{i}x=\omega_{i}\;\mathrm{for}\;\mathrm{all}\;i.$$

### 4.6. Other Algorithms

The CSSPI restoration algorithms summarized in this paper are widely used after consulting the relevant literature. However, there are a large number of recovery algorithms based on CS, which makes it a huge project to verify their performance in the field of SPI. Their provenance [143,144,145,146,147,148,149,150,151,152,153,154,155,156,157,158,159,160,161] is as follows: iterative soft threshold algorithm (IST) [162], fast iterative soft threshold algorithm (FIST) [163], approximate message passing algorithm (AMP) [164,165], least angle regression (LARS) [166], gradient projection for sparse reconstruction (GPSR) [167], FOCUSS solver [168], sparsity adaptive matching pursuit (StaMP) [169], and gradient pursuit (GP) [170].

Table 3 is a comparative summary of the several commonly used reconstruction algorithms in simulation, which may help to select reconstruction algorithms that meet different SPI systems.

## 5. Simulation

In terms of several aspects such as imaging quality, running time, and robustness to noise, to show their pros and cons, this section compared the performance of the above CSSPI measurement matrices and reconstruction algorithms on both simulated and experimental data. Without losing generality, we used “cameraman” with a size of 64×64 pixels as the test image in the simulation. “Cameraman” is a natural image with a continuous tone, which is extensively employed in MATLAB image databases and the digital image processing field.

Here, multiple experimental settings need to be clarified. (1) Concerning the quantitative comparison of image quality, we employ peak signal-to-noise ratio (PSNR) and structural similarity (SSIM) as metrics. (2) When the measurement matrix is compared with the results of the OMP algorithm, the number of iterations is set to 1/4 of the sampling patterns. (3) In comparison with the performance of the reconstruction algorithm, the iteration number of all greedy algorithms is set to 200.

### 5.1. Comparison of Measurement Matrix Performance

When testing the performance of the CSSPI measurement matrices mentioned above, we first selected DCT as a sparse dictionary. Subsequently, the OMP algorithm was adopted to reconstruct the image. Due to the fact that there is some uncertainty in the random measurement matrix, 10 times for each test, the average data were recorded.

**Figure 2:**
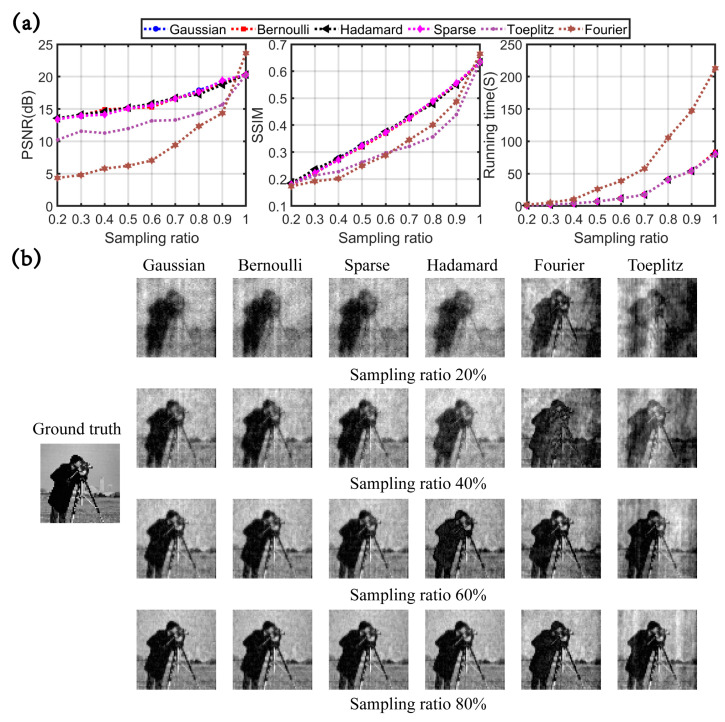
CSSPI results of different measurement matrices at different sampling ratios. (**a**) The three curve graphs show reconstruction PSNR, SSIM, and running time. (**b**) The reconstructed images under sampling ratios of 20%, 40%, 60%, and 80%.

**Figure 3:**
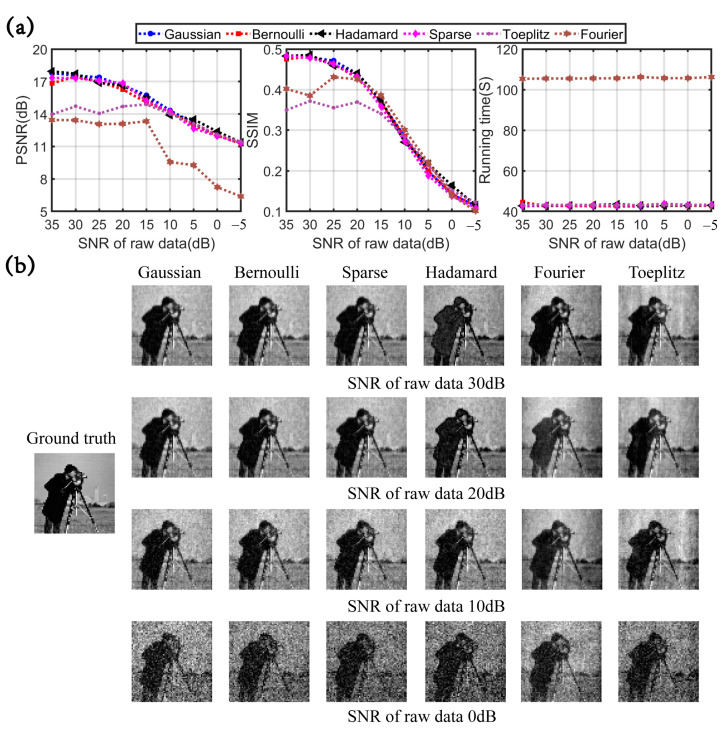
CSSPI results of different measurement matrices under different SNRs. (**a**) The three curve graphs show reconstruction PSNR, SSIM, and time. (**b**) The reconstructed images with SNR = 30 dB, 20 dB, 10 dB, and 0 dB.

The simulation results are exhibited in Figure 2 and Figure 3. Table 2 summarizes the six measurement matrices universally used in CSSPI. Figure 2a depicts the PSNR, SSIM, and running time under the sampling rate of 20%–100%. In the case of full sampling, the reconstruction quality of the partial Fourier matrix is better than that of other measurement matrices. Under sampling, the reconstruction quality of the partial Fourier and Toplitz matrices is worse than that of other measurement matrices. Apart from that, the Gaussian random matrix, Bernoulli random matrix, sparse random matrix, and partial Hadamard matrix show the same performance in terms of reconstruction quality. As conspicuously revealed in Figure 2a, the running time of the partial Fourier matrix is the longest. Figure 2b illustrates the reconstructed images of each measurement matrix at several different sampling rates.

Furthermore, different levels of Gaussian noise were used to pollute the measurements, and the image was reconstructed at a sampling rate of 80%. When the signal-to-noise ratio (SNR) is 35 dB to −5 dB, the performance of different measurement matrices is shown in Figure 3. The degradation rates of PSNR and SSIM values of Gaussian random matrices, Bernoulli random matrices, sparse random matrices, and Hadamard matrices are almost the same under different noise levels. The image reconstruction quality degradation rate of the Toplitz matrix and Fourier matrix is the lowest at low noise levels. Additionally, the PSNR and SSIM values of the Toplitz matrix are the same as those of all other matrices at high noise levels. Therefore, the Toplitz matrix and Fourier matrix show a better ability to suppress noise. In addition, the Gaussian random matrix, Bernoulli random matrix, sparse random matrix, and Hadamard matrix have the same ability to suppress noise. From the running time curve in Figure 3a, it can be seen that the reconstruction efficiency of all measurement matrices is not affected by noise. The above results can be further confirmed in Figure 3b.

### 5.2. Comparison of Reconstruction Algorithm Performance

In this work, we test the CSSPI algorithm mentioned above at different sampling rates and noise levels. It should be noted that not all algorithms are directly comparable because many algorithms make different assumptions about the measurement matrix at the beginning of their design. Therefore, to compare the performance of the algorithm in universality, the measurement matrix is the most widely used Gaussian random matrix, and the sparse dictionary is the DCT dictionary. At the same time, to reduce the influence of randomness on the results, the simulation of each algorithm is repeated 10 times, and the results are averaged.

In the Appendix A, we make an intra-class quantitative comparison and compare the mentioned algorithms in detail. From the result, we get several algorithms with better comprehensive performance, which are: OMP, IHT, BP, BPDN, TVAL3, BCS, and SB. Therefore, in the following comparison, we mainly take these algorithms as a representative and make an inter-class comparison of all kinds of algorithms in detail.

**Figure 4:**
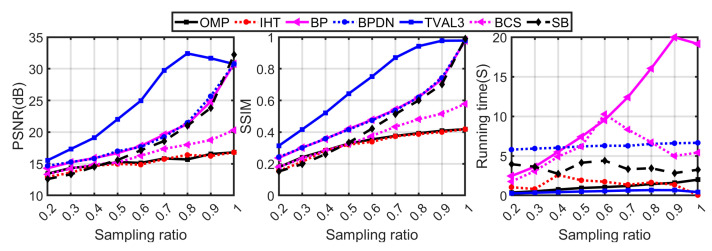
Quantitative comparison of various reconstruction algorithms at different sampling rates.

**Figure 5:**
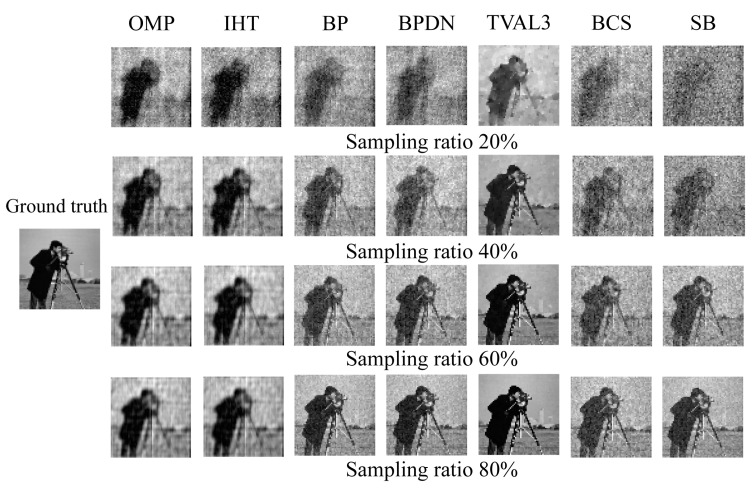
The “Cameraman” images were reconstructed by partial reconstruction algorithms at different sampling rates.

Figure 4 shows the results of an inter-class comparison of representative algorithms that perform better among various types of algorithms under different sampling rates. Figure 5 shows that partial algorithms reconstruct images at 20%, 40%, 60%, and 80% sampling rates, respectively. As can be seen from Figure 4, the reconstruction quality of the TV minimization algorithms is the best. This is because the image signal cannot be perfectly sparse even on a specific sparse basis, and the TV minimization algorithms were specially designed for the gradient sparsity of the image signal, so they are more suitable for image reconstruction. TVAL3 takes the least time to reconstruct the image. The reconstruction quality of the convex optimization algorithm is better than that of the greedy algorithm because the convex optimization algorithm looks for the global optimal solution in each iteration, while the greedy algorithm looks for the local optimal solution. In addition, the running time of the greedy algorithm is less than that of the convex optimization algorithm since the greedy algorithm only seeks the best matching atom rather than an atomic set in each iteration. The reconstruction quality and time of the Bayesian algorithm and Bregman minimization algorithm are between the convex optimization algorithm and the greedy algorithm.

**Figure 6:**
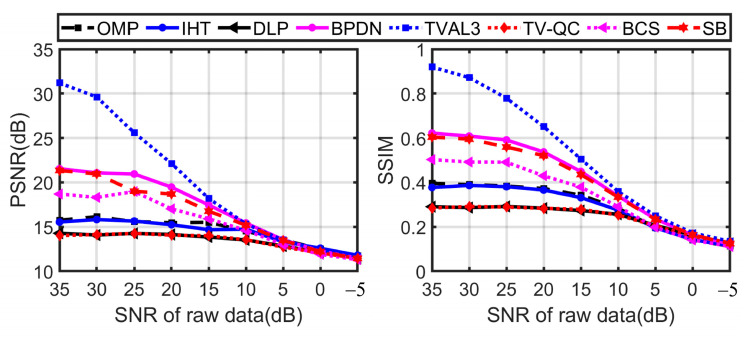
Quantitative comparison of various reconstruction algorithms under noisy conditions.

**Figure 7:**
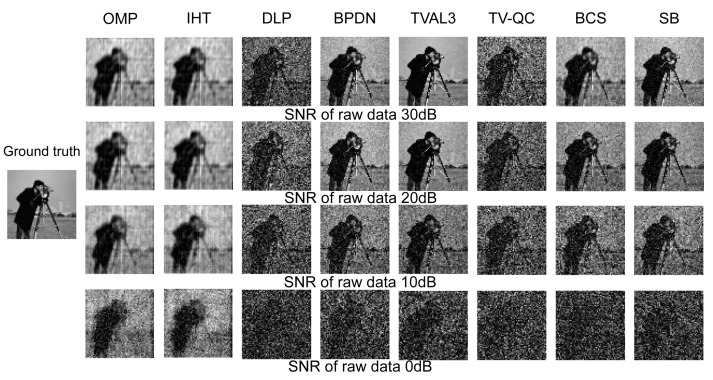
Comparison of the “cameraman” images reconstructed by different reconstruction algorithms from single-pixel measurements contaminated with Gaussian noise.

In the next test, we will further study the performance of various reconstruction algorithms with different noise. When the SNR is 35 dB to −5 dB, the performance of various reconstruction algorithms at a sampling rate of 80% is shown in Figure 6 and Figure 7. Figure 6 shows the results of the algorithms with better anti-noise performance in each group. It can be seen from the graph that the deterioration rate of DLP, TV-QC, and the greedy algorithm is the lowest, and the reconstruction result of TVAL3 is the best. The reconstruction quality of BPDN and SB is better than that of BCS and the greedy algorithm. It should be emphasized that for greedy algorithms, DLP and TV-QC, which have poor reconstruction quality in noiseless, they all have a low rate of deterioration in anti-noise, especially at a low SNR (that is, SNR < 10 dB), the reconstruction quality is equal to that of other algorithms. The PSNR of OMP and IHT is higher than that of all other algorithms under low SNR.

The same conclusion as a quantitative comparison can also be drawn from the reconstructed image shown in Figure 7. It can be seen from the figure that the reconstruction quality of the greedy algorithm is less affected by noise, and it is difficult to observe the difference under different SNRs. When the SNR is 0 dB, the four greedy algorithms can still observe the rough contours of the characters; however, other algorithms find it difficult to distinguish the contours of the characters in the image (such as TVAL3).

Based on the summary of the simulation test results of various algorithms, the TV minimization algorithm based on image gradient sparse shows the best comprehensive performance in various cases and is more suitable for image signal reconstruction. Table 3 is a summary of the simulation-tested CSSPI reconstruction algorithms in this paper.

## 6. Experiment

**Figure 8:**
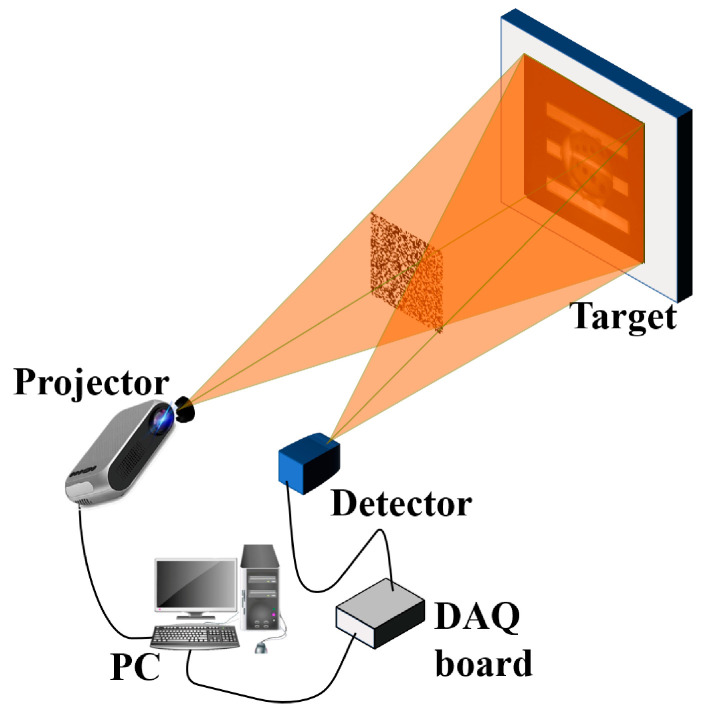
Schematic of the experiment setup.

**Figure 9:**
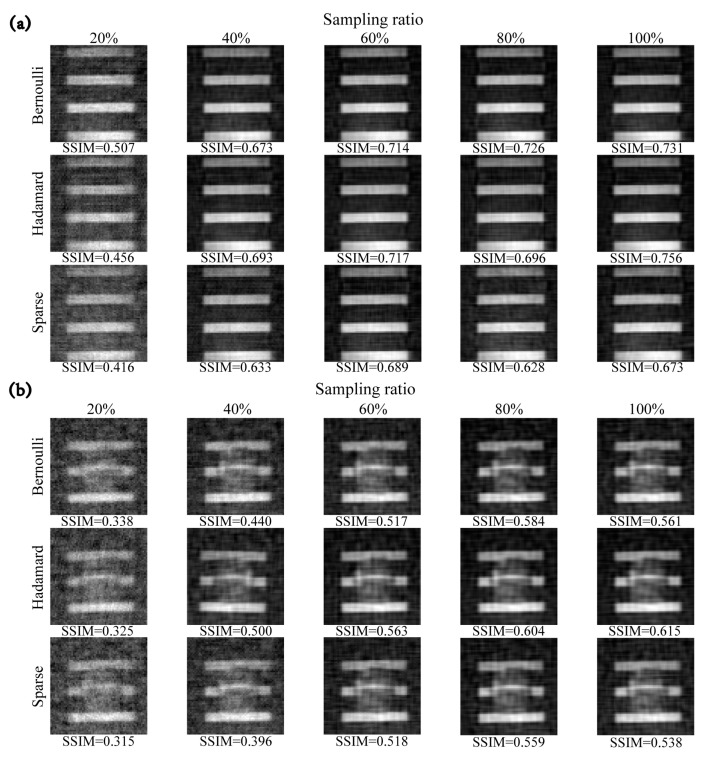
Comparison of images reconstructed by different measurement matrices at different sampling ratios. (**a**) Imaging results of “four bars”. (**b**) Imaging results of “ladybug”.

**Figure 10:**
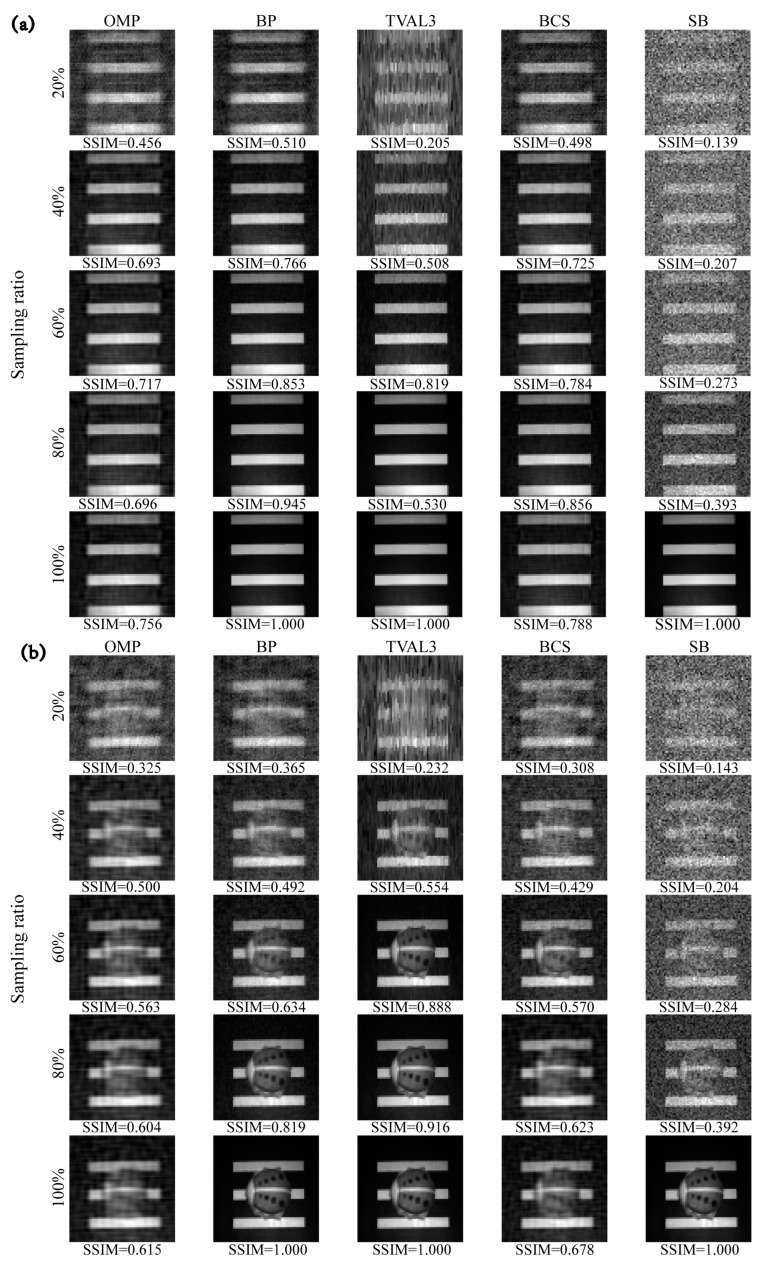
Comparison of images reconstructed by different reconstruction algorithms at different sampling ratios. (**a**) Imaging results of “four bars”. (**b**) Imaging results of “ladybug”.

In order to further compare the performance of different CSSPI measurement matrices and reconstruction algorithms, we designed a laboratory experiment to test the performance indicators under real experimental data, and the experiment setup is shown in Figure 8. A projector (ACER V36X) illuminates the target by patterns, and the reflected light of the target is incident onto a single-pixel detector (KG-PR-200K-A-FS) and transferred to the computer by the DAQ (NI DAQ USB-6216) for image reconstruction. In our experiment, we set the resolution of the projector to 1024×1280 pixels and projected a pattern every 0.3 s. This speed is very slow compared with the digital micromirror device (DMD), but the long acquisition time also means high SNR in the measurements. A simple picture of “four bars” and a complicated picture of a “ladybug” are used for the demonstration. The results are shown in Figure 9 and Figure 10. All measurement matrices are projected by differential projection to further reduce the impact of noise, and the imaging resolution is 64×64.

Figure 9 shows the reconstructed image results of unstructured random, partially orthogonal, and structured random measurement matrices (that is, Bernoulli, Hadamard, and Sparse) at different sampling rates. We select DCT as a sparse dictionary, and the reconstruction algorithm adopts the OMP. It can be found that the reconstruction quality of the sparse random matrix is slightly lower than that of the other two measurement matrices. At a sampling rate of 20%, the approximate outline of the target can be distinguished from the reconstructed results. For the “ladybug” model with more details, the quality of the reconstructed image is slightly worse. Additionally, the reconstruction quality of Hadamard matrices is slightly better than that of the other two kinds of matrices. The experimental results of the measurement matrix are consistent with the conclusions of the above simulation tests.

Figure 10 shows the image reconstruction results of the convex optimization algorithm, greedy algorithm, TV minimization algorithm, non-convex optimization algorithm, and Bregman minimization algorithm under different sampling rates. We use the partial Hadamard matrix as the measurement matrix and the DCT as the sparse dictionary. For the sparse “four bars”, the SSIM of the reconstructed image is 0.7 at a 40% sampling rate for OMP, BP, and BCS algorithms. For the “ladybug” with more details, the reconstruction performance of the TVAL3 algorithm is the best. The performance of the SB algorithm based on Bregman minimization in the actual imaging system is not satisfactory. The experimental results support the conclusion drawn in Section 5.

## 7. Discussion and Conclusions

The different CS measurement matrices and reconstruction algorithms used in SPI have certain tradeoffs in hardware implementation, acquisition efficiency, recovery efficiency, imaging quality, and noise robustness, as shown in Table 2 and Table 3.

In addition, with regard to CSSPI, the mutual containment relationship between imaging quality and imaging efficiency is the predominant factor that limits its application. To deal with this problem, the author believes that it is essential to further ameliorate the performance of CSSPI in the following aspects: (1) In the aspect of signal acquisition, most of the current methods directly adopt the measurement matrix to conduct the linear measurement. If we can first take into consideration the possible noise in the actual environment and introduce some local nonlinear operations in the measurement, we can expect to get more robust measurements. (2) In terms of image reconstruction, assuming that we can combine the sparsity of the signal to deal with the optimization problem, it is expected to get a better reconstruction effect. (3) Machine learning is adopted to optimize the measurement matrix and reconstruction algorithm of CSSPI at the same time to achieve the best match, which is expected to strike a balance between measurement efficiency and imaging quality. There have been some efforts to use machine learning algorithms to improve the performance of CS, which shows the strong impact of machine learning on CS [87,171,172,173,174].

In summary, we introduced the principle of SPI technology based on CS. Under different parameter settings, we subsequently tested and compared the main-stream measurement matrices and reconstruction algorithms in CSSPI on both simulated data and real captured data, including sampling ratio and noise. Afterward, we investigated the problems currently existing in CSSPI. Furthermore, we set forth the future research direction and development trend accordingly. Our work provides a comprehensive summary of conventional CSSPI and provides experience in the development and application of CSSPI.

## Figures and Tables

**Table 1:** A comparison between the work of this paper and the existing work.

Works	Content
Our works	-Reviews the concept of CSSPI. -Summarizes the main measurement matrices in CSSPI. -Summarizes the main reconstruction algorithms in CSSPI. -The performance of measurement matrices and reconstruction algorithms in CSSPI is discussed in detail through simulations and experiments. -The advantages and disadvantages of mainstream measurement matrices and reconstruction algorithms in CSSPI are summarized.
Existing works	Refs. [40,41]: Review the development of CS.
	Refs. [42,43]: Review the main measurement matrices in CS.
	Refs. [44,45,46]: Review the reconstruction algorithms in CS.
	Refs. [2,47]: Review the development of SPI.
	Refs. [48,49]: Review the algorithms of SPI. For CS, only the TVAL3 algorithm is involved.

**Table 2:** Analysis and summary of various measurement matrices in CSSPI.

Type of Measurement Matrix		Definition	Advantages	Disadvantages	References
Random Matrix	Gaussian	-Each coefficient obeys a random distribution separately.	-The RIP property is satisfied with high probability. -Fewer measurements and noise robustness.	-Large storage space. -Difficult to implement in hardware. -No explicit constructions.	[62]
	Bernoulli				[63]
Semi-deterministic random matrix	Toeplitz and Circulant	-Each coefficient is generated in a particular way.	-Easy hardware implementation and robustness. -Sparse random matrix retains the advantage of an unstructured random matrix.	-High uncertainty. -More measurements. -For a particular type of signal.	[67,68]
	Sparse random				[70,71,72,73]
Partial orthogonal matrix	Partial Fourier	-Some rows are randomly selected from the orthogonal matrix.	-It is fast to generate and easy to save. -Easy hardware implementation and robustness.	-Fourier matrix needs more recovery time and measurement times. -The dimension limit of the Hadamard matrix.	[47]
	Partial Hadamard				[65,66]

**Table 3:** Summary of various reconstruction algorithms in CSSPI.

Algorithm		Advantages	Disadvantages	References
Convex	Dantzig	-Fewer measurements. -Noise robustness.	-High computational complexity. -Slower, not suitable for large-scale problems.	[109]
	BPDN			[101,104,105,106]
	BP			[101,102]
	DLP			[107,108]
Greedy	OMP	-Easier to implement and faster. -IHT and CoSaMP can add/discard entries per iteration. - Noise robustness.	-A priori knowledge of signal sparsity is required. -The sparsity of the solution cannot be guaranteed. -More measurements.	[113,114,115,116,117]
	CoSaMP			[120]
	IHT			[122,123,124]
	SP			[121]
TV	TVAL3	-Preserves the sharp edges and prevents blurring. -Noise robustness. -TVAL3 and TV-Qc are faster.	-Not linear. -Not differentiable. -Dantzig selector is slower.	[142]
	TV-DS			[109]
	TV-QC			[108]
Non-Convex	BCS	-More sparse solution. -Faster, suitable for large-scale problems.	-Rely more on the prior knowledge of the signal. -High computational complexity.	[130,131,132,133]
	IRLS	-Fewer measurements than the convex algorithm. -Can be implemented under weaker RIP.	-High computational complexity. -Slower, not suitable for large-scale problems.	[128,129]
Bregman	SB	-Faster, noise robustness. -Small memory footprint.	-More measurements than the convex algorithm.	[137]

**Figure A1:**
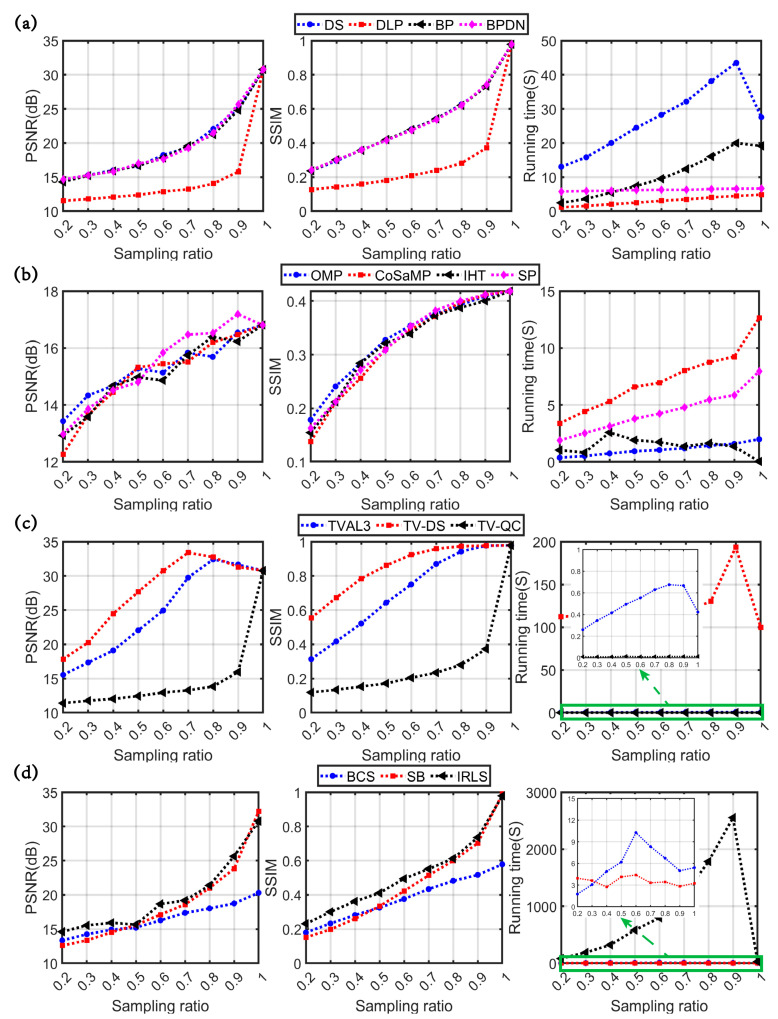
Quantitative comparison of various reconstruction algorithms at different sampling rates. (**a**–**d**) are the quantitative comparison results of the convex optimization algorithm, greedy algorithm, TV minimization algorithm, and non-convex optimization algorithm.

**Figure A2:**
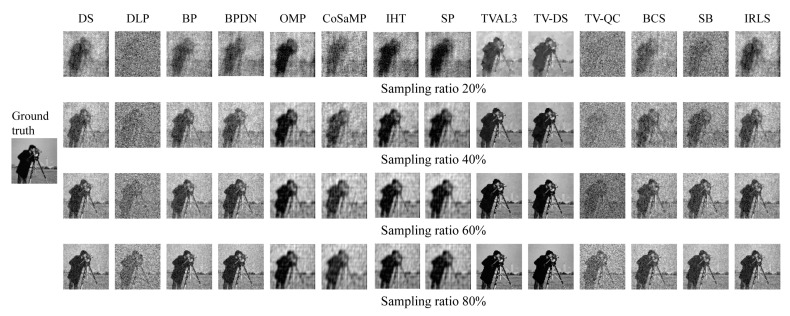
The “Cameraman” images were reconstructed by various reconstruction algorithms at different sampling rates.

**Figure A3:**
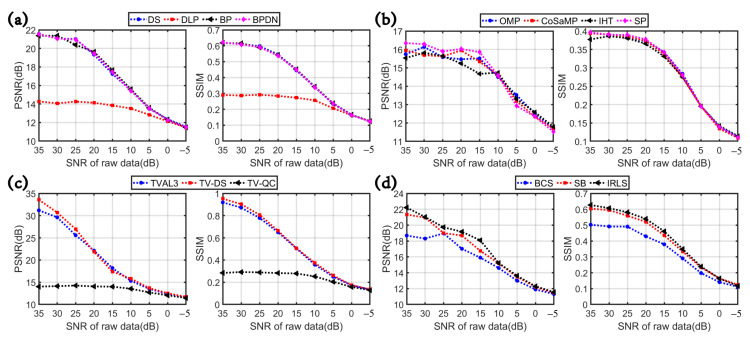
Quantitative comparison of various reconstruction algorithms with different noises. (**a**–**d**) are the quantitative comparison results of the convex optimization algorithm, greedy algorithm, TV minimization algorithm, and non-convex optimization algorithm.

**Figure A4:**
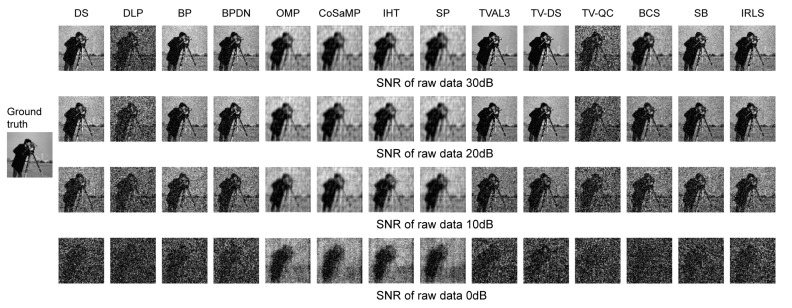
The “Cameraman” image was reconstructed by various reconstruction algorithms with Gaussian noise.

## Data Availability

Publicly available datasets were analyzed in this study. This data can be found here: [https://github.com/tyut-207/compressed-sensing, accessed on 8 May 2023].

## Appendix A

Here, we make an intra-class quantitative comparison and compare the mentioned algorithms in detail. We use “cameraman” with a size of 64×64 pixels as the test image in the simulation. The iteration number of all greedy algorithms is set to 200. Figure A1 shows the PSNR, SSIM, and running time curves at different sampling ratios, and Figure A2 is the reconstructed image at partial sampling rates. Figure A3 shows the PSNR and SSIM curves with different Gaussian noises, and Figure A4 shows the reconstructed images.

As can be observed in Figure A1a, the four convex optimization algorithms show similar performance; all of them can achieve perfect reconstruction at the full sampling rate. The reconstruction quality of DLP at a low sampling rate is weaker than that of the other three. In addition, the running time of BPDN and DLP algorithms is almost not affected by the sampling rate. The running time of the BP and DS is greatly affected by the sampling rate, especially the running time of the DS, which increases greatly with the increase in sampling rate.

As can be seen from Figure A1b, the reconstruction quality of the four greedy algorithms is almost the same. The PSNR and SSIM of OMP are slightly higher than those of other algorithms at the low sampling rate, while the SP is the highest at the high sampling rate. The running time of OMP and IHT is the least affected and is almost not affected by the sampling rate, while the running time of CoSaMP and SP increases with the increased sampling rate.

Figure A1c makes a quantitative analysis of three commonly used algorithms based on TV minimization, and the reconstruction quality of each algorithm improves continuously with the increased sampling rate. It should be noted that when the sampling rate is greater than 70%, the SSIM of TVAL3 and TV-DS tends to be stable, while the corresponding PSNR even decreases slightly. TV-DS takes much more time to rebuild than the other two algorithms; hence, to see the time spent by TVAL3 and TV-QC clearly, the green box area where they are located is locally enlarged. We find that the running time of TVAL3 and TV-QC is almost not affected by the sampling rate, even if there is a small increase in the range of less than 1 s. Therefore, in the reconstruction algorithm based on TV minimization, the reconstruction quality of using the Dantzig solver to solve the TV minimization problem is the best; however, it takes a lot of time, which is almost 100 times that of other algorithms. Although the reconstruction quality of the TVAL3 algorithm is slightly lower than that of the TV-DS, the SSIM value is more than 80% at a 70% sampling rate and takes little time.

From Figure A1d, we can see that the reconstruction quality of IRLS is better than that of BCS and SB at all sampling rates; however, it takes a lot of time, which seriously reduces its practical value, especially in real-time imaging scenes. The reconstruction quality of BCS is better than that of SB at the low sampling rate, and the running time is the least among the three algorithms. However, when the sampling rate is high, the comprehensive performance of SB is better. Therefore, BCS is more suitable for low sampling rate scenarios, while SB is suitable for high sampling rate scenarios.

When the SNR is 35 dB to −5 dB, the performance of various reconstruction algorithms at a sampling rate of 80% is shown in Figure A3 and Figure A4. From Figure A3a, we can observe that with the decrease in SNR of single-pixel measurement, the image reconstruction quality of the four convex optimization algorithms will decline. The decline rate in reconstruction quality in DLP is the slowest. The degradation rates of the DS, BP, and BPDN are almost the same. From Figure A3b, we can see that the anti-noise performance of the four greedy algorithms is the same; however, there is only a slight difference. From Figure A3c, we can see that in the algorithm based on TV minimization, the deterioration rate of TV-QC is the lowest. The reconstruction quality of TVAL3 is slightly worse than that of TV-DS in cases of high SNR. The reconstruction efficiency of TVAL3 is higher, so the comprehensive performance of TVAL3 is still the best. Figure A3d gives the anti-noise performance of the non-convex optimization algorithm and Bregman minimization algorithm. The two types of algorithms have the same deterioration rate under the condition of Gaussian noise, in which the reconstruction result of IRLS, which belongs to the non-convex optimization algorithm, is the best.
